# Men’s Refashioning of Masculine Identities in Uganda and Their Self-Management of HIV Treatment

**DOI:** 10.1177/1049732318823717

**Published:** 2019-02-14

**Authors:** Steve Russell

**Affiliations:** 1 University of East Anglia, Norwich, United Kingdom

**Keywords:** HIV/AIDS, gender, masculinity, illness and disease, experiences, coping and adaptation, self-care, adherence, Africa, qualitative, Uganda

## Abstract

Studies in sub-Saharan Africa show that masculine identities contribute to men’s relatively lower uptake of HIV services. Although useful, these studies pay less attention to men’s agency to negotiate and refashion masculine identities which better suit their lives as men living with HIV. In this article, I analyze the refashioning of masculine identities among men living with HIV in Uganda, adjustment processes which helped their self-management, and adherence to treatment. In-depth interviews with 18 men are thematically analyzed. Physical recovery was the embodiment of recovered masculinity and underpinned the men’s ability to refashion alternative, hybrid masculinities. Men negotiated and refashioned two forms of dominant masculinity already identified in this context, respectability and reputation, notably being a responsible father again and supporting other men with HIV, and being strong, resilient and an HIV survivor. Understanding men’s refashioning of masculinities can inform service providers’ approaches to reach more men with HIV treatment.

## Introduction

Despite gender structures which offer men numerous material benefits and decision-making authority, studies in sub-Saharan Africa over the last 10 years, including studies in Uganda, show that men face greater challenges than women when it comes to accessing HIV treatment services, adhering to drugs and continuing on treatment and care (Cornell, McIntyre, & Myer, 2011; DiCarlo et al., 2014; Mills, Beyrer, Birungi, & Dybul, 2012; Muula et al., 2007; Siu, Seeley, & Wight, 2013), with resulting higher mortality rates (Alibhai et al., 2010; Birungi & Mills, 2010; Braitstein et al., 2008; Cornell, Myer, Kaplan, Bekker, & Wood, 2009).

Masculinities and the related stigma affecting men living with HIV help explain these gender inequalities with service uptake (Camlin et al., 2016; Chikovore, Gillespie, McGrath, Orne-Gliemann, & Zuma, 2016; Fleming, Diclemente, & Barrington, 2016; Mburu et al., 2014; Nyamhanga, Muhondwa, & Shayo, 2013; Siu et al., 2013; Skovdal, Campbell, Madanhire, & Mupambireyi, 2011; Wyrod, 2011). Studies highlight a tension between widespread ideals and practices of masculine identity, such as physical strength, emotional and physical resilience, independence, authority over women and sexual prowess, and HIV service providers’ recommendations about what patients need to do and be to become “good” HIV patients and “therapeutic citizens” (Mfecane, 2011; Siu et al., 2013). HIV and its treatment can undermine masculine identities and consequently men are frequently less able to admit there is a problem, seek support or remain engaged with treatment.

The social research on masculinities and men’s use of HIV services is nuanced, capturing a diversity of masculinities and their fluidity over time and place. For example Siu et al. (2013) identified two interrelated dominant masculine value systems among Itheso gold miners in Uganda which influenced men’s performances of masculinity in different settings, including their HIV testing and treatment behavior. First, masculine respectability, encompassing the values and ideals of being a good husband, working hard to provide for the family and responsible fatherhood. The norms and values of respectability reflect those of dominant public institutions such as the church, family, and the state, and are public masculine ideals endorsed by women as well as men. Second, reputational masculinity, informed by the values shaped and endorsed among men in a given social domain, and the values by which a man’s status, honor, and manhood are judged by other men. Among the miners in Uganda these included physical strength (to be a tough miner), authority over women, sexual prowess, independence and control over decision making, and almost compulsory spending on leisure and drink.

Over the life course, men are also likely to shift their masculine identities (Coles, 2009), for example, Siu et al. (2013) found that older men were more reluctant to be tested due to the threat a positive result would pose to their respectability (as older men and husbands they should not be promiscuous). However, if the older men did get tested, masculine respectability values (being a responsible father) enabled them to become more committed to HIV treatment and stay well to support their families.

These studies, however, tend to emphasize the structural aspects of dominant masculinities, that is the obligations, norms, and informal “rules” which constrain men’s HIV treatment behavior, or sometimes enable it. They pay less attention, however, to men’s everyday practices and agency to change these very structures: to renegotiate masculine values which are dominant in their communities and live different masculine identities to suit their new lives and to manage well on antiretroviral therapy (ART). Only a few research studies have noted men’s agency to negotiate masculinities after they see the benefits of ART and physical recovery (Camlin et al., 2016; Dageid, Govender, & Gordon, 2012; Mfecane, 2012; O’Brien & Broom, 2013; Siu, Wight, & Seeley, 2014). More detailed evidence and conceptual analysis of this refashioning process, however, remain limited.

In this article, I thematically analyze evidence about men’s refashioning of masculinities in Uganda while on ART, referring to the notions of emergent and hybrid masculinities (Inhorn & Wentzell, 2011) or mosaic masculinities (Coles, 2009). I adopt the sociological approaches of structuration (Giddens, 1984) and habitus (Bourdieu, 1977) to emphasize people’s agency to reshape the very social structures which also pattern their behaviors. This brings to the fore men’s room for maneuver to refashion their notions of masculinity to better suit their new circumstances. These refashioning processes were important adjustments to the condition, the treatment regimen, and their sense of self: and so important adjustments to help them cope and effectively self-manage their long-term condition. Better understanding of masculine identities in relation to HIV can assist HIV service providers consider their own service delivery approaches for reaching more men with HIV treatment and care.

## Self-Management and Masculinities

Self-management of a long-term illness refers to the different work which people do to cope with and adjust to a health condition and its treatment, and to sustain well-being (Schulman-Green et al., 2012). The work is not just about taking medication but extends to emotional and psychological adjustments such as incorporating the illness and treatment into one’s identity (Russell & Seeley, 2010) and sustaining a positive self-image (Swendeman, Ingram, & Rotheram-Borus, 2009). Three broad domains of self-management work for long-term conditions, and more specifically HIV, have been conceptualized from systematic reviews of the literature (Schulman-Green et al., 2012; Swendeman et al., 2009). First is focusing on illness needs and managing physical health, for example, adhering to drugs, eating well, stopping drinking, and using condoms. Second is mobilizing resources, for example, disclosing, joining support groups, and building productive relationships with health care providers. Third is living with a chronic illness (psychological and social domains), notably adjusting to and normalizing the condition and adjusting to one’s new sense of self and identity.

The majority of HIV self-management instructions for patients in Uganda at the time of the study focused on the first domain of illness management (Russell, Namukwaya, Zalwango, & Seeley, 2015), particularly emphasizing adherence, stopping drinking, and changing sexual behavior. The implications of these messages in Ugandan society are not gender neutral because of existing gendered behaviors in this setting: In general, it is men who have to make more behavior changes with regard to drinking or their sexual behavior to become a “good patient” (Russell et al., 2015). As Mfecane (2011) argues, men must abandon their lifestyles and leisure if they are to become good “therapeutic citizens” (“These drugs deprive of us fun” [Mfecane, 2011, p. 129]).

Gender structure refers to the socially constructed and performed roles, obligations, responsibilities, and behaviors of sexed bodies in particular social contexts and structural situations (Martin, 2004). Masculinities are constructed in relation to femininities within this gender structure, and although they vary across societies, prominent features of masculinity which enable male social dominance include greater access to resources and wealth ownership, being the provider for the family, physical strength, and emotional resilience (Wyrod, 2011). Most dominant masculinities, what Connell (1995, 1998) terms hegemonic masculinities, legitimize men’s authority over women, so legitimizing patriarchy.

The concept of hegemonic masculinity enables analysis of a hierarchy of masculinities, and so not just power relations between men and women, but also between men (Coles, 2009; Connell, 1995). Hegemonic masculinities influence expectations and behaviors for men and women which contribute to the subordination of women, but which also operate to subordinate men who seek to live alternative (and subordinated) masculinities. In Uganda, Siu et al.’s (2013) work on respectable and reputational hegemonic masculine value systems and other studies in Uganda which have found similar hegemonic masculinities (Mburu et al., 2014; Wyrod, 2011) demonstrate how a serious illness such as HIV, which undermines many of the valued performances of manhood, poses serious challenges to men’s masculine identities. Masculinities then drive particular patterns of HIV stigmatization among men in Uganda (Mburu et al., 2014; Russell, Zalwango, et al., 2016; Siu et al., 2013; Wyrod, 2011) and hinder men’s uptake of HIV testing and treatment services.

Three aspects of the diversity and fluidity of masculinities are particularly relevant to this analysis of men’s refashioning of masculinities. First, a variety of hegemonic masculinities can co-exist in a given setting (Coles, 2009; Connell, 1995; Connell & Messerschmidt, 2005), among different groups based on intersections of class or occupation, gender, sexuality, ethnicity, and other subcultural identities. Put another way, a variety of social settings and identity groups exist which enable diverse dominant masculinities to be formulated and lived by different groups of men.

Second, masculinities as social structures are constantly in flux (Connell & Messerschmidt, 2005) because men relate to dominant masculinities in complex, creative, and transformative ways. Masculinities are constantly being shaped and refashioned as men live their lives, for example, during different phases of life, in response to new relationships, responsibilities, and, importantly for this article, to accommodate changes to health and physical bodies (Coles, 2009; Inhorn & Wentzell, 2011). The term “emergent” masculinities captures how masculinities are always in flux (Inhorn & Wentzell, 2011), are performances that are “ever in progress” (Inhorn & Wentzell, 2011, p. 803), as men live their lives in changing circumstances.

Third, and related to the above, Coles (2009) argues that at a broader theoretical level, hegemonic masculinity is often applied to the analysis of male power at a structural level, for example, how groups exert power over other groups to achieve dominant positions, but at the expense of men’s agency. For Coles (2009, p. 30), this structural emphasis means that “. . . the fluidity of masculinity is rarely given critical consideration in the context of men’s lives . . . ” Research on men’s adjustments to living with HIV and starting ART, for example, could avoid these structural limits by focusing on “. . . the strategies men use to negotiate masculinities in their everyday lives” (Coles, 2009, p.30). Faced with new circumstances, men have agency to fashion their own masculinities, drawing from a range of existing forms of masculinity to create what Coles (2008) terms “mosaic” masculinities, or hybrid masculinities. In this article, I examine men’s refashioning of these emergent and hybrid masculinities while on ART, identities which helped them to do the work of self-managing HIV and taking daily treatment.

Coles (2009) combines notions of hegemonic masculinity with Bourdieu’s (1977) theory of habitus, social field, and forms of capital to examine how men can deploy their agency to negotiate masculinities which suit their own circumstances. Habitus refers to a set of durable dispositions of thoughts and practices which shape, but which do not determine, people’s everyday lives (Bourdieu, 1977). People are collectively influenced by habitus, but at the same time the structures of habitus are constantly reshaped by people’s actions. Men draw on the habitus of various masculinities at their disposal, lead their lives according to circumstances, and do not necessarily or always have conscious purposes to reproduce or challenge the norms or values influencing their daily practices. But as men lead their lives with a new condition such as HIV, for example, many will be reconfiguring how they think of themselves and their male identity. Gidden’s structuration theory is a similar theorization of structure–agency interaction: social structures such as masculinities shape men’s lives, but their social practices will collectively both reproduce structures of masculinity, and also creatively alter these structures, leading to both social continuity and change.

These structure–agency interactions help us see how men, faced with HIV and the need to adjust to life on ART, can deploy their agency and refashion their masculine identities. They have room for maneuver within the habitus of masculinities. In new circumstances, men can select dispositions which better suit their new lives and “. . . choose to disassociate themselves from the mainstream and operate in social milieu where their masculinity is dominant in relation to other men” (Coles, 2009, pp. 30–31).

An earlier study in Uganda has shown how men’s decisions to begin ART are supported when they attend HIV treatment facilities which became part of a new social domain of supportive relationships with counselors, health workers, and fellow patients, where they acquired new knowledge and self-confidence (Russell et al., 2015). Health facilities become sites where men can reconceptualize or reframe HIV and themselves as men (Russell et al., 2015; Watkins-Hayes, Pittman-Gay, & Beaman, 2012).

In this article, qualitative findings are presented on men’s refashioning of their masculine identities, how these adjustments helped them remain on treatment, and what lessons this might offer for HIV service providers wishing to reach and retain more men on HIV treatment. The men were drawing on older forms of respectable and reputational masculinity to suit their new circumstances, and newer discourses of masculinity they had been encountering in the new HIV-ART social domain: they were, therefore, building “emergent” and hybrid masculinities to suit their new circumstances.

## Methods

In 2011–2012, qualitative and quantitative data were collected for a study on the ways that people living with HIV (PLWH) on ART in Wakiso District, Central Uganda, were coping with and self-managing the condition and adhering to treatment. The aim of the study was to develop understanding of the factors which enabled or hindered people’s ability to self-manage and adhere to treatment. In this article, the qualitative findings on men’s masculinities and self-management of treatment are presented.

Participants were recruited from the Entebbe branch of The AIDS Support Organization (TASO), the government hospital in Entebbe, and three government health centers with referral links to Entebbe hospital. To be eligible, participants had to have been on ART for more than 1 year. A list of eligible patients was compiled from each facility, the lists were stratified by age and gender, and 42 participants were then purposively sampled from the gender and age categories to ensure gender balance, a mix of ages, and a range of patient experiences. Four participants could not be interviewed successfully or more than once and were excluded from final analysis. Of the 38 participants included in the analysis, 18 were men.

Ethical approval for the study was obtained from the Uganda Virus Research Institute Science and Ethics Committee and the Research Ethics Committee of the University of East Anglia, United Kingdom. Overall, approval was granted by the Uganda National Council for Science and Technology. The data were anonymized using participant codes and pseudonyms. All participants provided written informed consent at the start of the research, and verbal informed consent was sought in each subsequent meeting.

Interviews were conducted by four experienced Ugandan social science researchers at the Medical Research Council (MRC), Uganda. The two male researchers in the team conducted the interviews with the male participants, who were interviewed twice. The first interview was a relatively unstructured interview about each participant’s life and illness history, conducted over two to three visits because of the wide-ranging and sensitive nature of some of the questions. These multiple visits allowed for iterative learning and the development of more focused follow-up questions. The first interviews were conducted in places chosen by each man, usually at their home but on a few occasions in settings further from home to avoid suspicion from neighbors and any involuntary disclosure of their HIV status. When conducted in the men’s homes, the MRC researcher also spent time with the man if he was working in the house, garden, or on other activities. The first interviews were not taped, because experience in this setting indicated people are more open when not being recorded, especially during the first few interviews. Interviews were conducted in the preferred language of the participant, in the majority of cases Luganda. Detailed notes from the interviews and observations were taken and were written up afterward in English.

The second interview was semistructured and recorded, transcribed, and then translated into English. The question guide was informed by the preceding life and illness history interview, as well as the research objectives and theoretical frameworks which informed the research. It explored fully participants’ approaches to self-management since becoming HIV positive and starting ART, for example, their feelings and responses to testing positive, questions of stigma and disclosure, their work and relationships, and their treatment seeking and adherence. The use of several visits allowed a degree of rapport to develop which led to rich discussions of participants’ experiences.

Qualitative data were initially organized into broad codes using QSR Software NVIVO 9. To check analytical rigor, two researchers independently coded these results. A more detailed thematic analysis was then undertaken (Green & Thorogood, 2004), using a mix of inductive and deductive approaches. Themes and subthemes were identified based on the narrative content itself, examining each transcript line and paragraph in detail, but also based on the research questions and existing theoretical and empirical work. In this way, the analysis was an iterative process, developing themes, and concepts from the data, then linking these to the existing literature (Green & Thorogood, 2004). The themes and subthemes developed for different areas of writing were then discussed in more detail by the team members and agreed after a 2-week analytical workshop. Themes were tested further by checking counter examples and exceptions.

Quotes used in the article are either the words of the participants or the interviewer’s words used in the write up of the first interview which was not audio-recorded. Frequently, repeated expressions across the interviews are not quoted but cited using quotation marks.

## Results

### Participant Characteristics

The majority of the men were in the middle phases of their life course and had children (9/18 were 30–39 years old, and 7/18 were 40–49 years old). The other two men were 51 and 74 years old. Most had been taking ART for 2 to 3 years, with the longest period on treatment being 8 years. Five of the men stated they were single and 12 were in relationships. They engaged mainly in small scale farming, fishing, and building trades, and a few were in the formal sector (policeman, teachers). Nearly half of the men were income poor (8/18 men), defined in terms of struggling to meet basic food needs. The others had an adequate material standard of living, through their salaries or successful trades. Those able to cultivate around their homes were usually able to eat one meal a day, but two men in extreme income poverty faced a daily struggle for enough food.

### Physical Recovery and Rebuilding Respectability

Bourdieu’s (1977) forms of capital are also applied by Coles (2009) to examine how men negotiate their status position within different social domains of masculinity. The possession of valued forms of capital by men in a given field of masculinity gives them status, creating hierarchies. In addition to the social, cultural, and economic capital which dominant groups seek to legitimize as valuable and possess, in the field of masculinities physical capital is also important: The male body and its strength, resilience, and image are a highly valued resource (Coles, 2009). Physical capital can enhance other valued forms of capital for men such as economic or social capital (e.g., their ability to work, status as a worker or position in their social networks).

The physical capital of the 18 men in the study had seriously deteriorated with HIV (all except one had become very sick, and some bed-ridden, before testing), undermining social capital (due to stigma), and valued economic capital (ability to work, earn and provide). Their subsequent recovery on ART meant that they recovered their physical capital and embodied masculinity, vitally important for the performance of valued masculinities. This embodied masculinity underpinned the men’s ability to rebuild their respectability, as well as reputation. Without physical capital, you could not rebuild respectability through work and providing for one’s children. Without it, if you looked sick, you were labeled as weak or immoral and could not be a respectable father or husband. And without physical capital, you would also struggle to fashion new forms of masculine respectability in the wider community as a man living with HIV, for example, acting as an inspiration and adviser to others. Body image was crucial for refashioning masculinities as men on ART.

Looking across the men’s narratives, they had constructed a clear chronological logic for getting tested, starting ART, and staying on ART, framed in terms of their masculine respectability and their responsibilities as a man, which can be distilled as the following sequence: I was weak and desperate to get better, and had two options, death or treatment; I was encouraged by seeing other men recover on ART; I decided to start the drugs, and got support from health workers; I started to feel better, to eat more, to become strong; I could go back to work, and therefore could provide for my family, as a man should.

Some of the men used the analogy of an “engine overhaul” when discussing their physical recovery, particularly the fishermen who used boat engines for their livelihood. Men took pride in their renewed strength and ability to provide for their families, embodying a return to a “proper” masculinity.

When people see him now, they exclaim that he has a powerful engine. This is because he is strong and can now work and do anything. These are the same people who were saying that he was going to die.

The men also expressed joy about their physical appearance, about looking good and “passing as normal” among people in the wider community (also reflecting a degree of ongoing fear of stigmatization). The minority who were more open about their status talked about this pride in their looks when with others, as this fisherman explained:

In the past there was so much fear (about HIV) . . . (but now) I drink my beer and I tell the people around that I am HIV infected, and I am proud . . . I show off because I look good.

Four interrelated processes (themes) of refashioning masculine respectability are examined below. A common thread through them was the new sense of responsibility that the men felt for their own health and that of the wider community.

#### A new sense of responsibility for my own health and the well-being of others

Daily drug adherence had become the most important new responsibility. The men’s renewed health showed them the benefits of adherence. Through the support and encouragement of counselors and fellow patients, and the new habits they were developing of taking the medication, the men’s dispositions toward health and risk-taking had changed. In the new social domain among others living with HIV, people often spoke about the drugs as “food,” something which just had to be consumed. One respondent used the analogy of a car (the drugs) and a driver (the patient): A responsible driver never has a problem, but if he drives recklessly, then he will die.

Such a shift to more respectable or responsible values and behaviors inevitably created tensions with the value systems of reputational masculinity, but the men appeared to be managing or negotiating these tensions. There was, for example, a selective “disobedience” of the health workers’ instructions which enabled a balance between new responsibilities and some of their earlier ways of having fun. The men had reduced their alcohol consumption, for example, but occasionally still went out with friends to have a beer for pleasure, which they thought was important to sustain their male friendships (and so important for their masculine identity and well-being). This was their negotiation of the terrain of masculinity to ensure their health *and* well-being were sustained.

It is hard for someone to stop eating the kind of food he grew up eating. . . . He cannot stop alcohol because when he sees a friend drinking, he also feels like drinking, although he does not drink everyday.

The men’s reprioritization of health was linked to their reappraisal of the value of life after they felt that they had “come back from the dead.” ART had given them a new chance for a future, one which included the chance to invest for their children’s future (Russell, Martin, et al., 2016). Men’s new sense of responsibility for themselves was therefore also relational, intertwined with a newly awakened responsibility for others, as a father primarily, but also as a husband and a fellow HIV patient what the men sometimes called a community of PLWH.

In their refashioning of respectable masculine identities, the men were drawing both from long standing notions of respectability, notably “working hard to provide for my family,” and newer values from the HIV-ART social domain. For some, HIV had been a “wake-up call,” and ART had given them a new sense of purpose as a father and provider, to “leave something behind”: “I work hard for my family, as a man should,” said one of the men. This responsibility was a cause of worry for many of the men. One said that the support for his children was straining him financially, but that “he had to face life as a man,” and their children were a powerful motivation for them to continue treatment:

. . . My children are a reason to fight for my life—so I can take care of them . . . (before HIV) I did not know how to save money or even budget, and used it for things that did not matter, but ever since I was told that I am HIV positive, I realised that I had to plan for my family and I am building four houses for rent to help me financially.

The men’s overarching logic of responsibility for adhering to ART, described earlier, was evident throughout these discussions, and for the men who were more economically secure this masculine responsibility was widened to the extended family, such as the children of deceased brothers and sisters. Two of the men who were open about their status in the community were proud of being seen to be providing for other households, a valued respectable (and reputational) achievement, especially because it was a public rebuttal to stigmatizing discourses about those with HIV being irresponsible or a burden on others.

The men also emphasized their newly found sense of responsibility for the health of their partners, and also the wider community of PLWH, having reflected on their own illness experiences and their conversations with people in the new HIV-ART social domain. Preventing HIV transmission to others and advising others were two main responsibilities of a man living with HIV, aspects of a new responsible and caring masculinity:

He said that all the children tested negative but his wife tested positive. He told me that his wife refused to take Septrin and also go for further treatment arguing that she was feeling strong. . . . He has been encouraging her together with the counsellors from TASO but in vain, but he is now feeling relieved that she is talking about seeking treatment.

As with alcohol use, with sexual behavior some of the (usually younger) men would, sometimes, disobey the “rules” about extramarital affairs or multiple partners, but the few who said they did occasionally sleep with another woman claimed that they always used a condom. The men were also concerned about (re)infecting their partner, but some argued they could not always use condoms with their partner because they had other priorities as men to father a child (also the wish of their partner). Pleasure remained another reason for not using a condom with a long-term (also positive) partner, as one man explained: “you realise that you are not enjoying, and sometimes I say that condoms were not meant for us and that is why I do not use them.”

In their narratives, the men were, therefore, mostly portraying themselves as a “good patient,” pursuing the ideals and practices of a good “therapeutic citizen” found in HIV treatment and care discourses (Mfecane, 2011). They could not, however, always follow the “rules” or advice from health workers, and often for good reason. Their negotiated form of therapeutic citizenship appeared to be a positive shift toward feeling empowered, recovering control, and contributing to a sense of greater well-being, alongside the obvious medicalization and disciplining of the self which arose from taking ART and following the instructions encountered at the clinic (Russell et al., 2015).

#### Becoming expert patients: Listening and learning in the HIV community

Studies in Uganda and South Arica have shown that men struggle more than women to mobilize certain types of resource for their HIV self-management, notably social support and building productive partnerships with health workers (Mfecane, 2011; Siu et al., 2013). In these studies, men viewed clinics as female spaces, and taking instructions from female health workers was seen as a nonmasculine practice and sign of weakness. The men in the study, in contrast, had managed to form positive relationships with male and female health workers and counselors, who had encouraged and helped them reconceptualize their situation. The quality of these relationships and the trust within them, built through the attention and care they had received, were key factors which had encouraged the men to keep coming back, and to change. One illustrative comment came from a boda-boda (motorbike taxi) driver: “. . . these two ladies (at TASO) offered us good counseling and they are really important to us”; and another from a fisherman:

There was a counsellor called Irene who came and told me that I should not worry because they were also infected. She told me that someone can live for over 30 years if they are eating well. . . . Following her instructions is what has gotten me here and I am very grateful to her.

Counseling by other people living with HIV, and the caring relationships which counselors and other health workers helped to develop with the men (compared to overly hierarchical ones), encouraged the men to listen, adapt, and engage with treatment:

Counselling has been very important to me. . . . The experience has been like a child waking up and going to school . . . and what they told me at first I still remember. Now I am like a professor, the three years they have been counselling me have been as if I was studying a degree.

The way our basawo (health workers) treat us, it is not the same as those who administer treatment for malaria or fevers. They treat us like people . . . they counsel us and they take care of us. They (health workers who give other treatment) are really difficult people . . . they even slap you.

In a few cases where a female health worker (at a government provider) was reported to have scolded a male patient, or staff had kept patients waiting, this particular group of men had been willing to swallow their pride or wait for the nurses: They were willing to play the game needed to keep obtaining the drugs they valued so much. Other men were reported to have stormed out, never to return.

#### Respectability within a new community of people living with HIV

Regular appointments at the clinic were a time and a space where a new collective identity was being fashioned through interactions with counselors, health workers, and among the patients themselves. The fashioning of a shared responsibility to tackle HIV and help or advise others were important processes in the men’s refashioning of responsible masculinities, as one of the research assistants reported: “The respondent added that the clinic is always crowded, but this gives them time to share their experiences and also give some new people advice on how to cope with HIV.” One man captured well this new sense of responsibility, fashioned in the social domain of the clinic:

When we are gathered at the clinic, we benefit a lot. This situation united us and we are the same. In fact, we call ourselves members; so when we meet, we simply greet hello member. It is as if we are in a club. You see now when we meet and we are conversing, one says that I still drink some alcohol and you also take the opportunity to tell them . . . that alcohol is bad you should leave it if you still need your life . . . and to continue with your plans.

The men who were open about their status sought to raise awareness and offer peer support for other men in their community, especially for local teenagers and work colleagues, just as they had been supported when they first thought about going for a test. They argued that they could only perform this role because they looked healthy—otherwise they would be shunned and not listened to. This role was transformative for some, offering a new purpose and sense of leadership among fellow workers, as the expert and living example which others could follow.

### Refashioning Reputation: Fashioning Hybrid Masculinities

What might be interpreted as reputational masculinities, the marks of honor and status among fellow men, were less frequently discussed in the interviews compared with issues of respectability or responsibility. This is not surprising, given that reputational masculinities are performed among peers, and the interviewers, although males, were MRC researchers who were perceived to be closely linked to the health care profession (and participants sometimes called them musawo, a Luganda term for health worker).

The men did, however, feel able to talk about reputational masculinities in two areas of their life. First, when talking about their “previous life,” which they said involved having a lot of fun, spending money on drinking and sleeping with women. Some reflected back with pride about their previous “conquests” over women, and one boasted how women loved him because he was “good at sex.” Others spoke with regret about their previous risk-taking. But both perspectives on life before HIV were narrated as a great contrast with their present life. Second, the men felt comfortable discussing aspects of their new lives which reflected a refashioned reputational masculinity: a hybrid of an existing habitus and new discourses of being a man who is taking ART. They would still go for a drink with friends, or some might have a brief affair (with condoms they said), and their renewed strength and looks helped them fit back into their previous social milieu. But they were also changing the meaning of what it meant to be strong, tough, brave, resilient, and a leader among men. Three interrelated processes are examined below. A common thread for these reputational masculinities was being a survivor and reclaiming control, by being smart, courageous, and up for the fight.

#### Being smart, adjusting, and coping

Preexisting and established markers of reputational masculinity for many of these men included being smart, canny, and successful (in business or with women, for example), and maneuvering around authority and dealing with challenges. Such canniness was now being applied to the skills of survival with HIV. Most men had been near death and proudly described how friends had exclaimed disbelief when they saw them healthy again. In their own minds, this gave them a new sense of reputation among their friends and work colleagues in terms of their resilience: They were too clever and strong for HIV, they were a survivor, and they had out-maneuvered death and were achieving things once again in life. One aspect of this skill was to listen and adapt: “Once a person who has had the experience (of HIV and recovery) tells you something, you listen to whatever you are told if you are wise.”

For these men, the route to success is to accept your status and do something about it. Then, if you are clever, you adjust and start taking the tablets (the first domain of self-management—see the introduction) and change the way you live so you can stay well (third domain of self-management). To refuse treatment “makes no sense”:

. . . I hear them talk about HIV infected people like they are already dead and useless in this world . . . (but) I know what I am *musawo* and I take my ARVs to have better health. One who does not know their status is the one who talks like that but when they get to know about their status, their attitude changes. Those who fail to change die quickly.

AIDS in the 90’s killed my elder brother and the way he died was very bad. . . . In late 1992, my other sister also succumbed to the same illness, but now . . . if I say I am infected they say . . . how come his hair is dark or how come he has no rash on his body. So in this era . . . the health workers have done a commendable job and people refuse for nothing to go and seek treatment.

#### Restoring order and regaining control

The dominant value system of reputational masculinity endorsed men who had control over their lives, and authority within their families. Adhering to ART meant they could begin reestablishing this control after serious illness had undermined it. This sense of rebuilding control stemmed from established ideals (also respectability-related) of being strong, working hard, earning money and being independent. In addition, the HIV-ART social domain offered new reputational ideals of control to live by: the men spoke of a new-found confidence, pride in their knowledge about their condition, a self-efficacy with managing it, as well as being a role model for others.

Restoring order and control involved resource mobilization (the second domain of self-management), especially disclosure to family and friends to get support, and building good relationships with health workers. The latter resource mobilization revealed the overlap between the refashioning of reputational and respectable masculinities. The men were refashioning ideas about what a man in control can do and be, which included making more responsible decisions for himself if he accepts advice and even follows a woman’s instructions. This partnership process was articulated well by a participant who talked about the sense of control he derived from health workers’ instructions:

I am in control of the illness when I respect what the health workers tell me to do. . . . That is why you see me build and going forward and when I look back, before I started on ARVs, I used to be disturbed by diseases. . . . But now, I do not have that thought. If you ask me my dreams now or what I am planning, I can tell you that I am going to plant a mango tree and I will be able to eat fruits from it.

#### Being strong, brave, a fighter

The men reaffirmed their existing reputational identities using language of bravery, determination, resilience, and strength of personality as a man living with HIV. They were applying these valorized attributes to their new ways of being a man in the HIV-ART social domain. Several men used the metaphor of fighting or being at war against the virus: “What pushed me to do that (advise youths about HIV) was the desire to fight the virus, let me call it fighting, and make sure it is reduced”; “When I swallow my drugs . . . I feel my heart has settled like when killing a snake, you hit it once, twice and by the third strike at it, you see it weakening. This is how I feel sometimes”;

When we went for a seminar, we learned and after learning . . . I became firm like a hero who has joined a war because this is like war, you cannot retreat. When I got to know that I am infected . . . I said to myself be strong . . . I became strong and now I do my work. (Man, age 43)

Others referred to their resilience: “I am tough, that is why I have survived and am not among the dead,” and another man captured well the fearlessness required for the fight:

I fear nothing because I am a man, I should not be afraid of anything. . . . I am strong because I accepted that I am infected with HIV with the support of the *basawo*. They gave me ARVs as treatment and therefore I have no reason to be afraid. When someone gives you an instrument like a shield to fight with in a war, do you say that I am afraid? You have to fight.

The above quotes illustrate the way the men used the valued masculine ideals of being a fighter, toughness and bravery as metaphors for their self-management or adherence work, and also in their work supporting others, of being at war against a deadly enemy.

### Negotiating Ongoing Challenges to Masculinity

There were, not surprisingly, tensions and challenges for the men as they adjusted to life with HIV, and sought to refashion the way they lived, their self-image and image among others, both in terms of respectability and reputation. First, most had to cope with the ongoing threats to respectability arising from their HIV status, in terms of the stigma they feared would be enacted if others discovered their status. Most of the men had only selectively disclosed their status, in fear of losing respect: They could not predict how people would react, feared gossip, and wanted to preserve their dignity in the community.

In this village, aaa! I cannot tell them, I remain here like a king, and I do not spend time sympathizing with myself, or that everywhere I pass worry about people thinking I am sick and I am going to die.

The majority adopted a nondisclosure strategy outside of the HIV-ART social domain and their immediate family, which they thought was the best way to preserve respectability and reputation in mainstream society with its own moralities and values: “I don’t want that (to disclose). I want them (my colleagues) to remain among my friends when I meet them . . . now my friends that I work with, they will know about it and start saying a lot.” A minority, in contrast, deliberately adopted a more open disclosure strategy, precisely because they wanted to fashion for themselves a new masculine identity in the wider community as a leader, an adviser, a fighter and survivor.

Second, despite recovering their health the struggle of rebuilding a livelihood returned to be the main challenge and source of worry for men. The processes of refashioning respectability and reputation in the field of work and being a provider were fragile. They also felt the added burden of wanting to recover economically what they had lost because of HIV. A constant feature of men’s narratives was the challenge of finding money, the struggle of poverty, and the need to rebuild, to be successful providers. One man, for example, the boda-boda driver, said he could not sleep peacefully, and another thought only about “dying without building a house for my children.” As one of the fishermen said,

Finances are low and so are the fish. School fees are so high and they put me under pressure. Sometimes I fear the situation in which I would leave my children because I do not want them to suffer. But I can sustain my family.

Third, and as Mfecane (2011) found in his study of men taking ART in South Africa, some men expressed an ambivalence about ART: They valued and adhered to ART, but it stopped their freedom and fun. A few men talked about how they felt deprived of the old ways of having “real” fun, like drinking, socializing with friends, and finding women:

I no longer go out for fun like I was doing before, because I have fun when I drink alcohol but I no longer drink like before. So when I see my friends going to have fun I do not follow them. I am not free to do many things because of the drugs because they have got several conditions. . . . I can say that this drug is the one that minimises fun.

In the process of negotiating their respectability (reducing drinking and sexual partners, using condoms), men were therefore also having to refashion their reputational practices. This was hard for those who, as a result of renewed health, felt the desire to have a good time again as they had done before with their friends. Some of the men had put the old partying behind them, but others managed to find a compromise between their desires for health and fun.

## Discussion

HIV can “demote” men to be lesser forms of men among their peers and communities (Wyrod, 2011). In this article, the data show that if men on ART can adjust and feel comfortable with their new masculine identities, this also helps them adjust to and better manage their condition. Seventeen out of the 18 men who participated in the study said they were adhering to ART and looked healthy in appearance. The men demonstrated adjustments to the condition and a new sense of self, and projected “. . . a positive view of the self and the world in the face of a health problem” (Swendeman et al., 2009, p. 1161). The method of using multiple interviews with each man, and observations in their home environments, gave confidence that these performances were not just for the interview but reflected more sustained and deeper adjustments to their outlook and identities. I do not claim that they were typical of many men in this setting who continue to struggle to get tested or adhere to treatment, but it is from such men that we can learn about adjustments to masculine identities which enable better engagement with HIV treatment, and so perhaps insights for ART interventions.

Men’s fashioning of masculine identities was relational, the product of a multitude of interactions in a new HIV-ART social domain. Structuration processes were also evident as men continued to refer to and reproduce dominant masculinities in this setting, but were also creative, shaping newly emergent and hybrid norms, values, priorities, and dispositions as men living with HIV. Health workers and peers living with HIV were part of this process, providing knowledge, support, and encouragement for this difficult process of reconceptualizing or reframing their situation (Russell et al., 2015; Watkins-Hayes et al., 2012).

The men had constructed a logical narrative for engaging with HIV treatment: physical recovery means strength, and so an ability to work again, which means I provide for my family as a man should, which brings me back respectability in the community and builds my self-esteem. The centrality of the body for practicing gender identity was evident (Inhorn & Wentzell, 2011; Martin, 2004). Bodily integrity was the embodiment of a moral and rejuvenated masculine integrity, and physical capital was important for men to rebuild other economic, social, and cultural capital needed to begin refashioning respectability and reputation. Health providers as well as the wider media (e.g., television, road side advertising boards) should therefore continue to develop innovative and eye-catching images and messages which emphasize physical recovery and performance of masculinities (Shand, Thomson-de Boor, van den Berg, Peacock, & Pascoe, 2014), as well as through peer role models who can visibly show through their own bodies the benefits of treatment (Mazanderani, Locock, & Powell, 2012).

Men’s alternative dispositions and practices were not always based on deliberate strategies for change. Rather, they reflected a gradual process of adjustment, of shifting priorities and behaviors in a new HIV-ART social domain surrounded by others doing similar things (or not, and them seeing the health consequences). Sometimes, however, the men did talk about a more purposive resistance to the prevailing stigmatizing narratives about men with HIV, and this process of resistance thinking and speaking, even in the interview itself, could in itself have been contributing to the refashioning of their identities.

A subtle hierarchy of masculinities was also perhaps emerging within the new HIV-ART social domain, linked to performances of respectability as “responsible” men living with HIV, and reputational performances as a fighter and survivor of HIV. Performances of an emergent responsible masculinity, such as adhering, stopping drinking, not infecting others, and advising others, were clearly partly performances for the interview, but as noted above, appeared to be consistent with their evident health and sense of pride and self-esteem. In contrast, references were also made to men who were not finding or had not found the inner strength to overcome fear and stigma, or the resources to be successful at the new work of survival, and so were heading for death, or had already died. The participants were therefore refashioning emergent dominant masculinities in their new social domain. They were no longer living “subordinated” lives in an environment of HIV stigmatization. As Coles (2009, p. 33) notes, men living masculinities which are not dominant in the wider society can still “assume a dominant position in relation to other men.” However, it should also be noted here that the men’s categorization of “those who are canny, fight and succeed” (Us), and those who “refuse to get tested, or struggle to adhere, or die” (Them), did not appear to be expressed as a means to divide the community of men living with HIV, or subordinate others, but to express their understanding of the challenges that many men face with self-management, such as stigma and poverty.

World Health Organization (WHO; 2016) has advocated differentiated service delivery (DSD) for HIV services and a move away from a “one size fits all” approach. Research on masculinities in different contexts can inform such differentiated services for men within national HIV programs. The groups often listed for more support are key populations groups, adolescents, and mobile or migrant populations, but there is also a need for services to be tailored to groups of men with specific barriers to treatment engagement. The WHO recognizes that “(i)nnovative service delivery models are essential to improve men’s access to HIV care services” (WHO, 2016, p. 254) and highlights the importance of peer support to help men navigate the health system and enhance adherence counseling. However, emphasis tends to be on the types, location, providers, and frequency of services. These are of great importance, but the building of relationships and trust which oil the service delivery machinery also need strong support (Gilson, 2003). The findings in this article indicate that DSD pathways would need to go beyond dealing with the location of services, to address questions of masculine identity, and so include counseling services which can support their work to renegotiate masculinities and related stigma.

The emergent and hybrid masculinities identified among men taking ART in this part of Uganda suggest three broad and interrelated approaches for ART interventions to better reach out to men. First is the need to ensure geographical accessibility so that men can access a new HIV-ART social domain and the trained health workers who can, potentially, play important roles in initial reframing and refashioning processes. Other studies have shown the importance of flexible opening hours to accommodate men’s work routines, for example (Camlin et al., 2016; Mills et al., 2012), decentralizing services to reduce the direct and indirect costs of care, and the use of mobile clinics or clinics close to busy work and transport hubs (Kuwane, Appiah, Felix, Grant, & Churchyard, 2009).

Second, in the understandable drive to prioritize ART access, more emphasis also needs to be placed on developing interpersonal quality of HIV services if they are to be acceptable and effective (Moyer & Igonya, 2014). The quality of counseling and support for participants appeared to have been critical to their ongoing engagement with ART treatment and the complex work of refashioning identities. Good relationships with health workers, and notably counselors, build trust and receptiveness to their messages (Mukumbang, Marchal, Van Belle, & van Wyk, 2018; Russell, Martin, et al., 2016; Shand et al., 2014). The gap between the life-worlds of men and the logic of clinical treatment needs to be bridged through listening, understanding, and empathy. Men’s refashioning of masculinities was a difficult terrain to navigate, particularly to negotiate conflicting habitus for how a man should have fun and be independent, compared with how a good patient should behave. Interactions with providers which offer an understanding of men’s options to continue enjoying themselves, but in different ways or on a more limited scale (e.g., less drinking), are likely to reap greater success than strict or dismissive approaches to their needs. This would also acknowledge the patient’s need to adopt an identity different to that of being HIV positive, to occasionally escape its responsibilities (Mfecane, 2011).

There are obvious resource and structural causes for poor interpersonal quality care. But in various fields of health care, communication is an area which can be worked on relatively easily and cheaply (Penn, Watermeyer, & Evans, 2011). Simple training methods for communication and sharing decision-making can start a conversation and begin change, for example, showing video testimonies of men’s bad and good experiences with HIV services, through role-play exercises, or even using actors and vignettes to stimulate discussion and change (Prose, Brown, Murphy, & Nieves, 2010). Given the values of masculinity which hinder men from admitting failure, or which mean men are likely to feel particularly belittled if a female health worker (who comprise the vast majority of health workers in these settings) scolds him for failing to take his drugs, improved interpersonal quality of care and communication will benefit all patients but might have a disproportionate impact for men.

Third, greater gender sensitivity within ART programs is needed to support men’s work to refashion masculinities. HIV stigma and barriers to treatment uptake are experienced differently by men and women (Russell, Zalwango, et al., 2016; Wyrod, 2011), and so the values of masculinity which promote health-seeking behavior could be better harnessed (Dageid et al., 2012; Mburu et al., 2014; Mfecane, 2012; Siu et al., 2013), such as promoting ART as a means of rebuilding strength and the ability to provide, as seen in this study. This would also require new training and practices among health workers, to enable them to work more sensitively and effectively with men, to accommodate their fears and needs, and to discuss how to move forward (Mfecane, 2011). A more radical approach would be to find additional, innovative and low-cost ways to offer livelihood support for patients, which can have a particular appeal to masculinities and for men as they seek to (re)build livelihoods (Camlin et al., 2016; Mburu et al., 2014; Siu et al., 2013). An example of low-cost support for livelihood development is the SEARCH project in Uganda, which linked treatment engagement with small lottery incentives (Camlin et al., 2016).

Initiatives might also support men’s refashioning of masculinities through gender transformative interventions. Gender structures are deeply embedded but are always changing. Studies are beginning to show that gender transformative interventions which get men to discuss, reflect on, and challenge masculine norms and gender inequalities, in social spaces and among peers groups which allow men to discuss vulnerabilities, for example, can help them refashion masculinities and reduce violence or risk taking (Campbell, Nair, & Maimane, 2007; Lynch, Brouard, & Visser, 2010; van den Berg et al., 2013; Wyrod, 2011). Only one study has examined how such gender transformative programs (“One Man Can” in South Africa) affect the uptake of HIV treatment (Fleming, Colvin, Peacock, & Dworkin, 2016): The results were positive. How best to design and implement these complex interventions to change social structures will vary depending on the context.

The complexity of the above interventions means they are likely to face challenges in achieving their goals, but involving peers in both health service delivery improvements and gender transformative interventions is often an ingredient for success (Fleming, Diclemente, & Barrington, 2016; Kuwane et al., 2009; van den Berg et al., 2013; Wyrod, 2011). Peers are more likely to be socially close to patients and can offer role models of change, for example, with respect to newly emergent and hybrid masculinities which are motivational for other men. In this study, most of the men spoke about the HIV clinic as a supportive space where both counselors who are HIV positive and fellow patients helped them reconceptualize their situation and adjust to HIV and life on ART (Russell et al., 2015). The use of peers or expert patients by TASO Uganda for the provision of decentralized HIV services partly explains the organization’s success in providing a supportive environment which has sustained retention in care (Abaasa et al., 2008; Okoboi et al., 2015). With the large number of people needing HIV treatment services in Uganda, DSD can help release human resources, while the use of peer supporters in care delivery can help contribute to less hierarchical relationships, providing good quality care which supports men, and women, to refashion their identities and sustain self-management.
